# Profiles or Continua? Reproducible but Redundant Creativity-Resource Configurations in PISA 2022

**DOI:** 10.3390/jintelligence14090207

**Published:** 2026-09-01

**Authors:** Faye Antoniou, Mohammed Alghamdi

**Affiliations:** 1 Department of Educational Studies, National and Kapodistrian University of Athens, 15784 Athens, Greece; 2 Department of Self-Development Skills, King Saud University, Riyadh 11451, Saudi Arabia; mhalghamdi@ksu.edu.sa

**Keywords:** creative thinking, creative ability, individual differences, psychological measurement, latent profile analysis, class enumeration, incremental validity, cross-validation, PISA 2022

## Abstract

Assessed creative thinking is a cognitive individual-difference construct—related to, but not equivalent to, conventional intelligence—that PISA 2022 measured directly at scale. We asked how its associated resources are structured, and whether discrete student “types” or continuous dimensions better predict creative-thinking performance. Using 44 education systems (306,704 students; complete-indicator sample 235,240), we estimated senate-weighted Gaussian mixtures of nine within-system standardized creativity indicators and related them to performance (10 plausible values; Fay balanced repeated replication). A six-profile solution was reproducible given adequate optimization (mean adjusted Rand index 0.95 across 12 subsamples), yet no class count was selected—information criteria improved monotonically from 2 to 12 classes—and the profiles were predictively redundant: in leakage-safe cross-validation they explained less variance (*R*^2^ = 0.028) than the nine continuous indicators (0.044) or a flexible continuous model (0.068) across *K* = 2–12 and under hard or soft classification. After a validated classification-error correction, performance-proximal dispositions and curiosity, more than relational perceptions, predicted creative-thinking performance. For measuring and modeling creative ability, continuous resource dimensions were more informative than discrete types, which amounted to an enumeratively underdetermined, predictively redundant re-description of continuous variation.

## 1. Introduction

Creative thinking is increasingly treated as a broadly distributed educational outcome (Beghetto & Kaufman, 2007; J. C. Kaufman & Beghetto, 2009), defined by the joint requirements of originality and effectiveness (Runco & Jaeger, 2012; Sternberg & Lubart, 1996). PISA 2022 administered creative thinking as a direct cognitive assessment for the first time in an international large-scale study (Barbot & Kaufman, 2025; OECD, 2023, 2024b). Its correlates are heterogeneous: creative self-efficacy, intellectual and artistic openness, imagination, curiosity, and supportive family and school contexts are all theoretically relevant (Allen et al., 2018; DeYoung et al., 2007; Gruber et al., 2014; S. B. Kaufman et al., 2016; McCrae, 1987; Tierney & Farmer, 2002). A common analytic response is to ask whether these resources cluster into qualitatively different student configurations.

Person-centered methods such as latent profile analysis (LPA) answer that question by modeling a finite mixture of subpopulations with distinct multivariate means (Hickendorff et al., 2018; Masyn, 2013; Spurk et al., 2020). Their appeal is communicative: naming a few “types” is more interpretable than reporting nine partial regression coefficients. But three questions must be answered before a profile solution can be interpreted substantively, and they frequently are not. First, is a class count selected—do fit indices favor a solution, or do they improve without bound, as often happens in large, non-normal samples where diagonal-covariance mixtures absorb skewness and unmodeled correlations (Bauer & Curran, 2003)? Second, is the chosen solution *reproducible* across random starts and subsamples? Third, and most importantly, do the profiles add *incremental value*—do they predict outcomes better than the continuous indicators from which they are built, out of sample? This study addressed these three questions for creativity-related resources, and only then asked whether different favorable configurations differed in performance.

### 1.1. Creative Thinking as a Cognitive Individual-Difference Construct

Directly assessed creative thinking is best understood as a cognitive individual-difference construct: a graded, measurable ability that varies between people and predicts performance on tasks requiring original and effective responses (Runco & Jaeger, 2012; Sternberg & Lubart, 1996). It is situated alongside, but is not equivalent to, conventional intelligence. Divergent-thinking and originality abilities have long been located within models of the structure of intellect and of human cognitive abilities (Carroll, 1993; Guilford, 1967), and creative-thinking performance is empirically related to fluid and general intelligence while remaining separable from it—a relation typically described as moderate and possibly threshold-like (Batey & Furnham, 2006; Jauk et al., 2013; Kim, 2005; Nusbaum & Silvia, 2011; Silvia, 2015). PISA 2022’s direct assessment therefore offers a rare opportunity to study creative ability as an individual-difference outcome in a large, cross-national sample, while recognizing that its scores partly index general cognitive engagement and academic skill (Kraus et al., 2026). Framing the construct this way sharpens the measurement question at the center of this paper: are the personal and contextual resources associated with this ability best represented as discrete student types or as continuous dimensions—and which representation better predicts creative-thinking performance?

### 1.2. Dispositional and Contextual Resources

Individual dispositions are robust correlates of creative outcomes: creative self-efficacy (Beghetto, 2006; Tierney & Farmer, 2002, 2011), openness/intellect (Feist, 1998; S. B. Kaufman et al., 2016; McCrae, 1987), and curiosity, which motivates the exploration that creative tasks reward (Berlyne, 1954; Gruber et al., 2014; Kashdan et al., 2004; Silvia, 2008). Contextual resources are also implicated: supportive family environments (Gute et al., 2008; Harrington et al., 1987), school belonging (Allen et al., 2018; Goodenow, 1993; Osterman, 2000), and autonomy-supportive teaching (Reeve & Jang, 2006; Ryan & Deci, 2000). Recent construct-validity work cautions that PISA’s creative-thinking scores partly reflect general academic skill and effort, and that self-reported creativity is only weakly related to performance (Kraus et al., 2026)—an important consideration when interpreting associations with self-reported resources.

### 1.3. Configurations, Equifinality, and Cross-System Caution

Developmental systems perspectives motivate attention to configurations and to *equifinality*—different antecedents converging on similar outcomes (Cicchetti & Rogosch, 1996; Sameroff, 2010). Equifinality is a developmental claim; cross-sectional data can at most show whether qualitatively different configurations coexist and whether they are associated with similar contemporaneous performance. We therefore use “configuration” descriptively. Cross-system comparison adds a caution: response styles, translation, and item functioning can differ across systems (Cen et al., 2026; Rutkowski & Rutkowski, 2025), so we standardized indicators within system and weighted systems equally, treating recurrence as a hypothesis to be tested.

### 1.4. The Present Study

We asked: (a) whether a class count is *selected* by the data; (b) whether the six-profile solution is *reproducible* across starts, subsamples, and independent per-system estimation; (c) whether profiles add *incremental predictive value* beyond the continuous indicators, out of sample and across resolutions; and only then (d) whether substantively different favorable configurations are associated with equivalent or merely above-average performance, and (e) which resources are associated with performance in variable-centered and within/between-school models.

## 2. Materials and Methods

### 2.1. Data Source and Participants

PISA assessed samples intended to be representative of each participating education system’s 15-year-old students, using a stratified two-stage design (OECD, 2024c). The public student questionnaire file contained 613,744 students from 80 education systems; the one system commonly counted among the 81 PISA 2022 participants but absent from this file is Ukraine, whose 2022 data were released separately. Of these 80 systems, 63 in this file had creative-thinking plausible values (of the 64 that administered the assessment; OECD, 2024b). Systems were eligible when at least 500 students had a creative-thinking plausible value and at least 50% coverage was available for every indicator; 44 systems met this criteria (listed in Appendix A). The 19 excluded creative-thinking systems all fell below the 50% coverage rule because they did not field the creativity-questionnaire scales (coverage ≈ 0 for most). The analytic sample was composed of 306,704 students in 11,620 schools (50.4% female; 12.5% immigrant background; ESCS *M* = −0.24, *SD* = 1.07). Because 76.7% of these students had complete indicator data, the complete-indicator sample (*N* = 235,240) was the primary analytic sample and the available-data model a sensitivity analysis (Table 1). Excluded (incomplete-indicator) students performed 0.48 *SD* lower on creative thinking and were lower in ESCS (Section 3), so the complete-case sample is positively selected; we therefore reproduced the principal outcome conclusions with the available-data classifications.

### 2.2. Measures

Nine OECD weighted-likelihood-estimate indices represented creativity-related dispositions and contexts (Appendix A Table A1). These IRT-scaled indices derive from multi-item questionnaire scales; item content, item counts, and per-system scale reliabilities are documented by education system in the PISA 2022 technical report (OECD, 2024c). Because the study used the OECD-derived index scores, the underlying item responses—which are available in the public database—were not loaded for the present analysis; the absence of student-level item-invariance testing is noted as a limitation. Each index was standardized within system using unweighted moments and then Winsorized at ±4 *SD*, in that order; within-system standardization removes each system’s location and scale so that values represent standing relative to same-system peers. Within-system moments were computed unweighted because the quantity of interest is each student’s standing relative to same-system peers in the achieved sample, and because PISA’s within-system samples are approximately self-weighting, leaving unequal inclusion probabilities little scope to distort these moments; a sensitivity analysis that instead standardized using senate-weighted within-system means and standard deviations recovered the same six-profile solution (mean Tucker congruence = 0.998). Two composites were formed as unit-weighted means of standardized indicators and then restandardized within the system so that their coefficients were comparable per standard deviation: creative agency (creative self-efficacy, openness to intellect, imagination, openness to art) and Relational Support (creative family environment, creative school environment, belonging, teacher support); curiosity was the ninth indicator. Creative-thinking performance was used to inform the 10 plausible values, each standardized within system and analyzed collectively (von Davier et al., 2009). Covariates were ESCS, gender (female = 1), and immigrant background. The nine indicators and the plausible values were standardized within the system; ESCS was retained on its original PISA scale (internationally standardized, *SD* ≈ 1) and gender and immigrant background were binary. Coefficients for the resource composites and curiosity are therefore expressed in within-system standard deviation units.

The two composites were formed for parsimony and interpretability, not as empirically established unidimensional latent variables. The four creative-agency indicators were moderately and consistently intercorrelated (*r*s = 0.37–0.53; mean *r* = 0.45; *α* = 0.76), consistent with a coherent openness-to-experience/creative-self dimension of the kind long implicated in creativity and in models of cognitive ability (DeYoung et al., 2007; S. B. Kaufman et al., 2016; McCrae, 1987). The label “agency” is a convenient shorthand: the composite deliberately blends relatively stable dispositions (openness to intellect and to art, imagination) with a self-referential efficacy belief (creative self-efficacy), and we retain it as an analytic summary rather than a claim that these four indicators index one causal trait. The four relational indicators were more weakly and heterogeneously intercorrelated (*r*s = 0.15–0.46; mean *r* = 0.26; *α* = 0.59) and combine four conceptually distinct sources—the family environment, the school’s institutional climate, class-level belonging, and the student–teacher relationship. The relational-support composite is therefore best read as a parsimonious analytic summary of these sources rather than a single latent construct; its lower internal coherence anticipates the opposing indicator-level associations reported below (see Table A3 in Appendix A), and all substantive claims about relational resources are made at the indicator level. The inter-composite correlations were 0.31–0.42.

### 2.3. Analytic Strategy

Mixture model and weighting: A K-component diagonal Gaussian mixture, $f(y_{i})=\sum_{k}\pi_{k}\prod_{j}\phi (y_{ij}|\mu_{kj},\sigma_{kj}^{2})$, was estimated by expectation maximization. All mixture estimation used senate weights: for student i in system c with final weight $w_{ic}$,

$$w_{ic}^{S}=\frac{w_{ic}}{\sum_{i\in c}w_{ic}}\cdot \frac{N}{C},$$

so that each of the C systems contributed total weight N/C and the weights summed to N; here, N is the pseudo-likelihood sample size entering the information-criterion penalty (not the Kish effective sample size). The primary estimand therefore gives each of the C education systems equal influence, rather than weighting systems by the size of the student population they represent; coefficients should be read as system-averaged associations, and population-weighted modal contrasts are reported as a sensitivity analysis. Each of the 80 Fay replicate weights was senate-rescaled by the identical formula. The available-data sensitivity model evaluated each student’s likelihood over the observed indicators only. This is a full-information (FIML-type) treatment that, unlike listwise deletion, retains partially observed students and yields consistent mixture parameters under a missing-at-random assumption—that is, if conditional on the observed indicators (and covariates) the probability of missingness does not depend on the unobserved indicator values. It is more parsimonious than multiple imputation but rests on the same MAR premise; because incomplete-indicator students scored lower on the outcome, we treat the close agreement between the complete-case and available-data solutions (below) as the key evidence that this selection did not distort the substantive conclusions. Sample code is shown in the Supplementary Material.

Class enumeration and reproducibility: Enumeration (*K* = 2–12) used a system-balanced sample (≤2000 students per system; pseudo-likelihood N = 88,000), with 10 random starts for *K* = 2–3, 25 for *K* = 4–5 and 7–8, 50 for *K* = 6, and 7–15 for *K* = 9–12. All fits used up to 250 EM iterations with a relative log-likelihood tolerance of 10^−6^; across *K* = 9–12, 37 of the 38 fits converged before the iteration cap (median 106–181 iterations by *K*), so the monotone improvement in the information criteria does not reflect unequal optimization effort across *K* (per-fit iteration counts are logged in tables in the Appendix A). We computed the Akaike and Bayesian information criteria on this unit-mean-weighted scale, entropy, and the proportion of starts that recovered the best log-likelihood within 5 units (in the balanced enumeration sample). Six profiles were retained as a provisional working resolution because the smallest class remained above 10% only through *K* = 6, whereas *K* ≥ 7 produced classes below 5% that appeared to subdivide existing profiles; these 10%/5% thresholds and the judgment that later classes were subdivisions are heuristics, not data-selected rules. Reproducibility was assessed by (a) the adjusted Rand index (ARI; Hubert & Arabie, 1985) between full-sample classifications from 12 repeated balanced subsamples (best of six starts) and the pooled solution; (b) recurrence of the outcome ordering across those subsamples; (c) independent per-system LPAs, matched to the pooled means by the optimal permutation and summarized by Tucker’s congruence coefficient averaged across matched classes then systems (Lorenzo-Seva & ten Berge, 2006); and (d) agreement in classifications and prevalence between the complete-case and available-data solutions. The repeated subsamples were drawn independently and could overlap; they were not independent replications.

Incremental value (leakage-safe cross-validation): To avoid the optimistic bias of preprocessing before splitting (Moscovich & Rosset, 2022), the entire pipeline was repeated within each fold of a repeated (3 × 5) cross-validation with folds assigned at the school level within system. Within-system standardization moments (for indicators and plausible values) were estimated from the training fold and applied to the validation fold; the mixture was refitted on training students only, using the best of four random starts (the fold maximum was recovered by a mean of 2.9 of 4 starts within 5 log-likelihood units); validation students were assigned by the training-fold parameters; outcome models were fitted on training students (senate-weighted) and used to predict validation outcomes, with weighted validation *R*^2^ using the validation students’ population weights. Prediction models comprised only the resource representation (no covariates), so the comparison isolated the value of discretization: the nine continuous indicators, a flexible continuous model (the nine indicators plus their nine quadratic terms and all 36 pairwise interactions—54 predictors in total, fitted by unpenalized weighted least squares), and the profiles (as modal dummies and, separately, as posterior class probabilities). Fold *R*^2^ was averaged across the 10 plausible values. Because folds sharing training data are dependent, we summarized results as the mean of the three repetition-level means and the range across the 15 folds, not as an inferential interval; *negligible* incremental value was defined as an incremental cross-validated *R*^2^ below 0.005 relative to the flexible continuous benchmark; this criterion was fixed before the relevant cross-validation comparison was examined (the study was not preregistered, so “a priori” here means analyst-declared rather than registered). The substantive conclusion did not hinge on this exact cutoff: the observed increment (0.004) was an order of magnitude smaller than the flexible model’s advantage over the profiles (0.068 − 0.028 = 0.040), and the profiles never exceeded even the nine linear indicators; the increment would be labeled non-negligible only under a stricter 0.0025 threshold, and was clearly negligible at 0.010. 

Distal outcome models: Because classification error remained non-trivial in the working six-profile partition (complete-case sample; entropy = 0.755; mean maximum posterior probability = 0.832), distal outcomes were estimated primarily with a BCH classification-error correction (Bakk & Vermunt, 2016; Bolck et al., 2004; Vermunt, 2010), which outperforms uncorrected modal assignment for continuous distal outcomes; modal and posterior probability-weighted estimates are reported for comparison. In the BCH step the classification matrix D with $D_{st}=P(\mathrm{modal}=t|\mathrm{class}=s)$ was estimated from the posterior probabilities, and each case’s correction weights were taken from row $W_{i}$ of $D^{-1}$; the implementation was validated against a synthetic benchmark with known class means, which it recovered to within 0.02 *SD*. The classification matrix was well-conditioned (condition number = 1.5; determinant = 0.31; correct-classification diagonal 0.74–0.89); as is expected for inverse-classification (BCH) weighting, the resulting case weights were signed and bounded (range −0.15 to 1.38), with no extreme or degenerate values, indicating a numerically stable correction. The adjusted model (configuration dummies + covariates), pairwise contrasts among the favorable configurations, and two one-sided equivalence tests against a 0.10-*SD* smallest effect size of interest (SESOI; Lakens et al., 2018)—chosen a priori as a small effect; the study was not preregistered—used BCH weights and the 80 senate-rescaled Fay replicate weights (k=0.5). For each plausible value the within-imputation BRR covariance was computed and the estimates and covariances were pooled across the 10 plausible values by Rubin’s (1987) rules,

$${\mathrm{Var}}_{BRR}(\hat{\theta })=\frac{1}{80(1-k{)}^{2}}\sum_{r=1}^{80}({\hat{\theta }}_{r}-{\hat{\theta }}_{0}{)}^{2},\;\;T=\bar{U}+(1+1/10)B.$$

These standard errors were conditional on the estimated partition; adjusted models used 224,573 students with complete covariates (removed listwise). The population-weighted sensitivity used uncorrected modal assignment.

Within- and between-school decomposition: To examine whether resources were associated with performance at the school level, each composite was decomposed into a school mean (between-school) and a within-school deviation, and performance was regressed on both, with covariates, senate weights, and per-plausible-value BRR. School means were the student-final-weight-weighted means within each school (median 20 students per school; 10.7% of schools had fewer than five students, for which the between component equals the school value and the within component is near zero). Because between-school associations may reflect socioeconomic composition, a sensitivity model also decomposed ESCS into within- and between-school components (see Table A4 in Appendix A). This is a fixed within/between decomposition, not a random-effects multilevel model, and its coefficients are described as associations in this specification.

### 2.4. Robustness

We compared class-varying versus equal within-profile variances by BIC and examined within-class residual correlations (Bauer & Curran, 2003); re-derived eligibility at 70%, 80%, and 90% coverage; and reran the profile solution excluding Albania (the one eligible system annotated for creative-thinking non-comparability; OECD, 2024b) and, separately, the seven eligible systems annotated by OECD (2024b) for sampling-standard cautions (Australia, Denmark, Hong Kong [China], Jamaica, Latvia, the Netherlands, New Zealand). Analyses used reproducible Python 3.14.7 code with fixed seeds.

## 3. Results

### 3.1. Is a Class Count Selected? No

**Figure 1:**
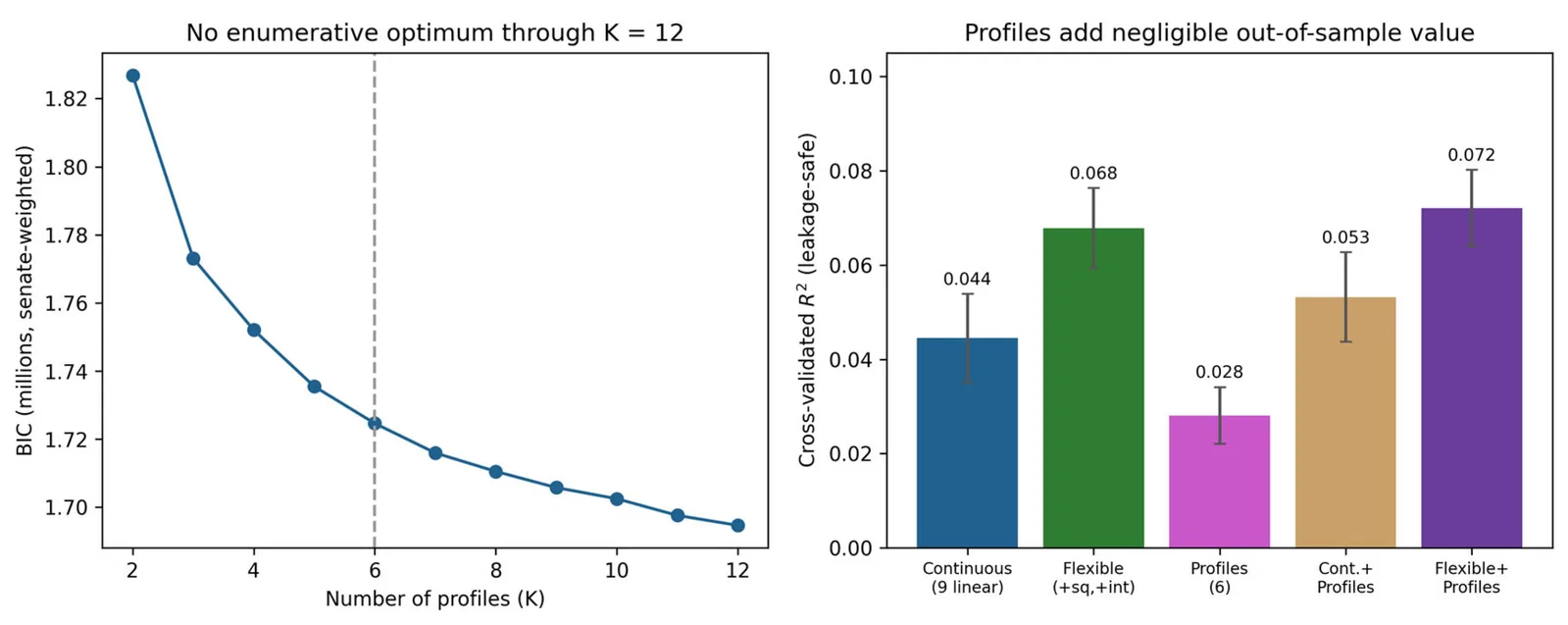
No class count is selected, and profiles add negligible out-of-sample value. (**Left**) senate-weighted BIC continued to improve from 2 through 12 profiles, so no enumerative optimum emerged. (**Right**) leakage-safe, school-grouped cross-validated *R*^2^ (best of four starts per fold; error bars = *SD* across 15 folds); the six profiles predicted worse than the continuous indicators and contributed marginally to a flexible continuous model.

The Akaike and Bayesian information criteria improved monotonically from two through twelve profiles (Table 2; Figure 1, left), so no defensible enumerative optimum emerged within the tested range, and entropy remained ≤ 0.75 in the balanced enumeration sample and generally declined while the smallest class shrank to ~1% by *K* = 12. Consistent with distributional flexibility rather than a genuine class count, class-varying variances were strongly preferred over equal variances (BIC 5.20 M vs. 5.47 M on the full complete-case sample), although within-class residual correlations were modest (max |*r*| = 0.22, mean 0.06).

### 3.2. Given Adequate Optimization, Is the Six-Profile Solution Reproducible? Yes

In the balanced enumeration sample, the best six-profile log-likelihood was recovered (within 5 units) by 60% of 50 random starts, with comparable behavior at *K* = 5, 7, and 8. Once located, the solution was highly reproducible: across 12 repeated balanced subsamples, the mean ARI to the pooled classification was 0.95 (*SD* = 0.03, range 0.88–0.97), and the complete-case and available-data solutions agreed closely in classifications (ARI = 0.92) and prevalence (Table 3), not merely shape (Tucker φ = 0.998). Independent per-system LPAs recovered the pooled configurations moderately (mean congruence = 0.83, range 0.63–0.97), only weakly related to system sample size (*r* = 0.26). Excluding Albania or the seven sampling-caution systems left the solution essentially unchanged (Tucker φ = 0.999–1.000; ordering preserved).

### 3.3. Do the Profiles Add Predictive Value? Negligibly, at Any Resolution

**Figure 2:**
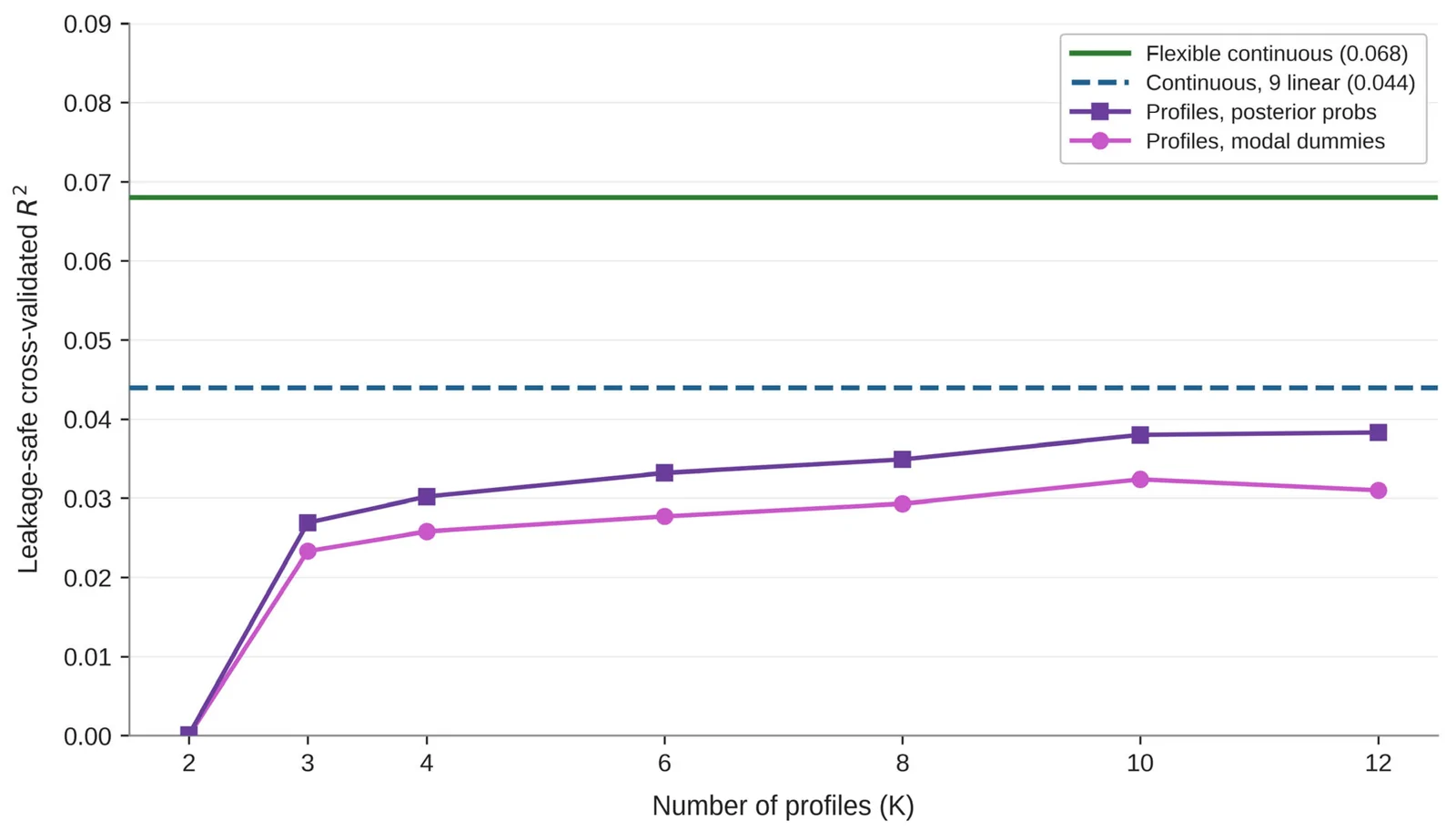
Profile prediction never approaches the continuous model across the evaluated resolutions. Leakage-safe cross-validated *R*^2^ for profile prediction at *K* ∈ {2, 3, 4, 6, 8, 10, 12} (a lighter sensitivity analysis: three folds, one start per fold), with profiles entered as modal dummies or posterior class probabilities, against the continuous-model benchmarks (horizontal lines). No evaluated resolution, and neither hard nor soft classification, approached the flexible continuous model.

In leakage-safe, school-grouped cross-validation—refitting the mixture (best of four starts) and standardization within each training fold—converting the continuous indicators into profiles discarded predictive information (Table 4; Figure 1, right). Out of sample, the six profile dummies explained *R*^2^ = 0.028—less than the nine continuous indicators (0.044)—and a flexible continuous model explained 0.068. Adding profiles to the nine linear indicators raised *R*^2^ from 0.044 to 0.053, and adding them to the flexible model raised it from 0.068 to 0.072; the mean incremental *R*^2^ of profiles over the flexible model was 0.004 (repetition-level means 0.004, 0.004, 0.004); below the 0.005 threshold was defined a priori as negligible. This pattern held at every resolution evaluated from *K* = 2 to *K* = 12 and whether profiles entered as modal dummies or posterior probabilities (Figure 2): the best profile prediction (posterior probabilities at *K* = 12) reached only *R*^2^ ≈ 0.038, which was still below the nine linear indicators (0.044) and well below the flexible model (0.068); at *K* = 2 profile prediction was ≈ 0. The profiles’ small increment was consistent with discretized nonlinearities and interactions that a continuous model captures directly; consistent with this, the nine indicators were not unidimensional (first principal component = 35.8%; second eigenvalue = 1.31).

### 3.4. The Configurations and Their Outcomes

**Figure 3:**
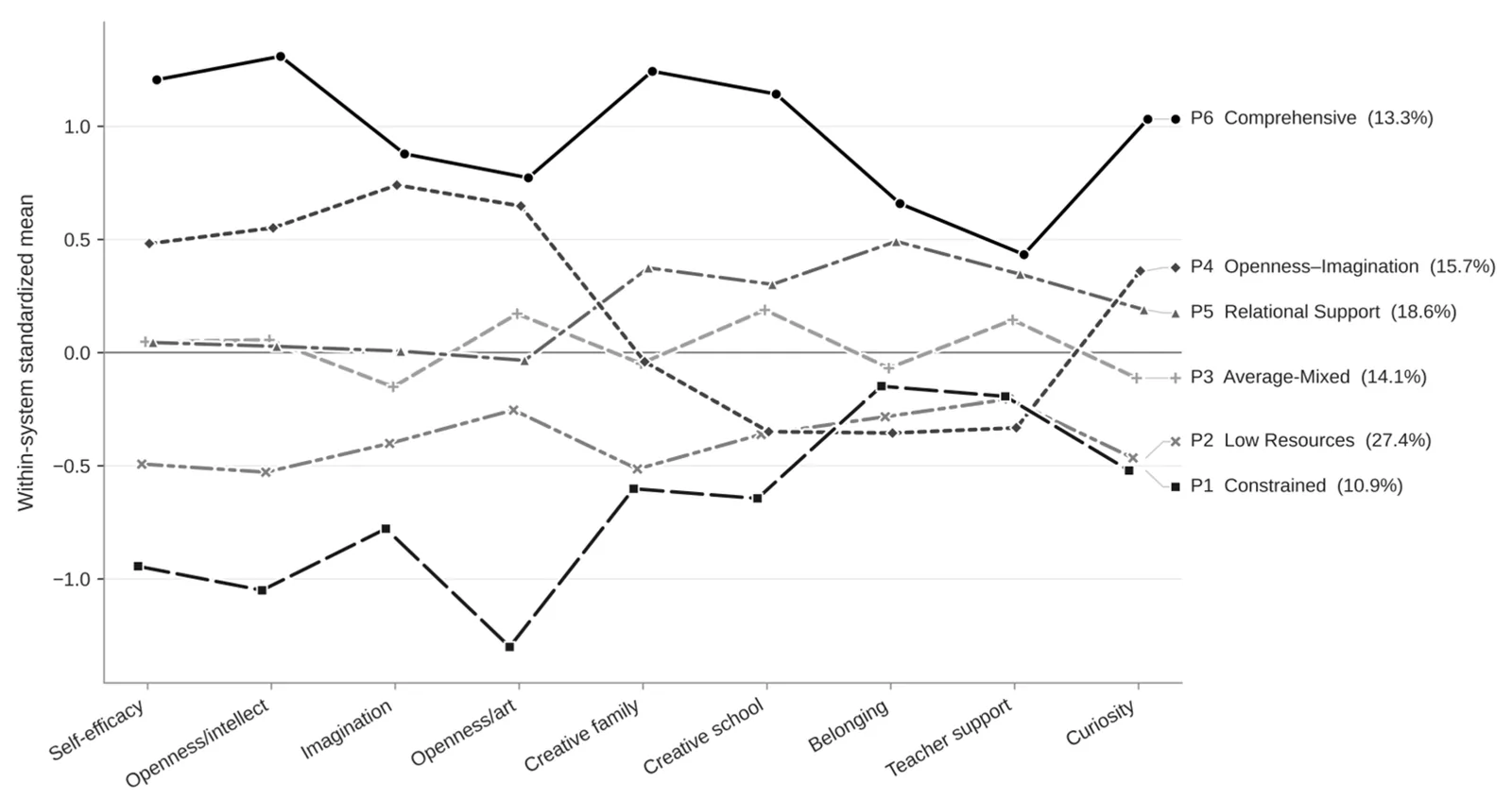
Within-system standardized means for the six configurations (complete-case sample). Legend percentages are senate-weighted posterior prevalence. Openness–Imagination is high on dispositions and low on context; Relational Support shows the reverse. Profiles are shown for description; no class count was selected (Table 2) and they add negligible out-of-sample value (Figure 3 and Figure 4).

**Figure 4:**
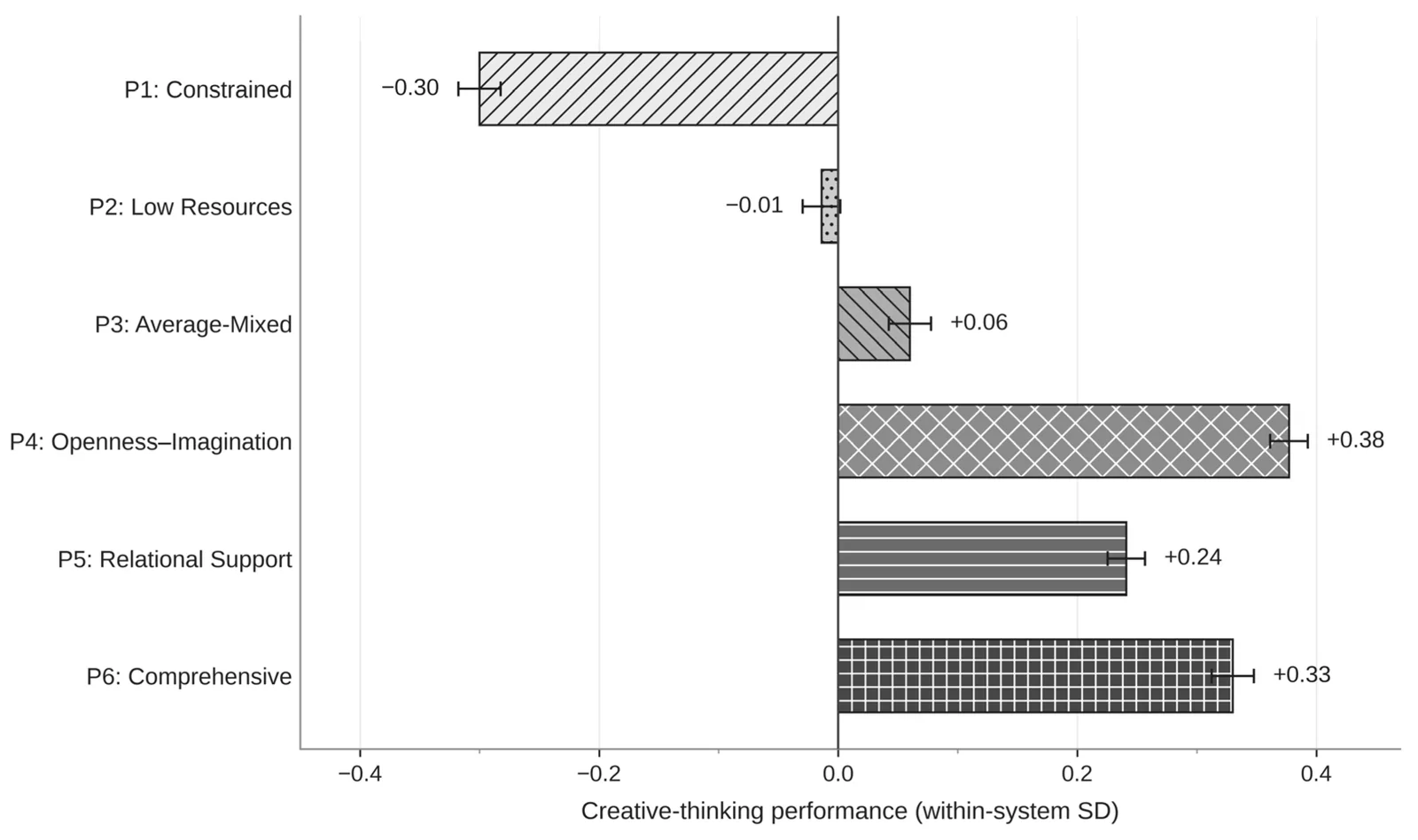
Creative-thinking performance by configuration. Bars are mean modal-class performance pooled across 10 plausible values, standardized within the system; error bars are 95% conditional Fay BRR confidence intervals. The ordering with Openness–Imagination highest recurred in 12 of 12 subsamples; adjusted, BCH-corrected contrasts appear in Table 4 and Table 5.

For description, the six-profile complete-case solution (Figure 3; Table A2 in Appendix A) comprised Constrained (10.9%), Low Resources (27.4%), Average-Mixed (14.1%), two crossing configurations—Openness–Imagination (15.7%: high dispositions, below-average context) and Relational Support (18.6%: near-average dispositions, above-average context)—and Comprehensive (13.3%). The three resource-favorable configurations showed substantially above-average performance (Figure 4); Average-Mixed was marginally positive (0.06 *SD*). The ordering with Openness–Imagination highest recurred in 12 out of 12 subsamples. After covariate adjustment with the BCH correction, the three favorable configurations again exceeded Average-Mixed (Table 4). The adjusted contrasts were 0.295 for Openness–Imagination, 0.226 for Comprehensive, and 0.182 for Relational Support. This ordering was robust to the estimator. Uncorrected modal contrasts (0.233, 0.146, 0.206), population-weighted modal contrasts (0.208, 0.132, 0.174), and posterior-probability-weighted distal means (0.357, 0.330, 0.230) all preserved it. In the available-data sample the adjusted modal contrasts were similar (0.203, 0.176, 0.123 for Openness–Imagination, Comprehensive, and Relational Support), so the selective complete-case sample did not distort the contrasts.

**Table 5:** Adjusted, BCH-corrected creative-performance contrasts (conditional Fay BRR, complete-case sample).

Term	*b*	*SE*
Intercept (P3)	0.017	0.012
P1 Constrained	−0.329	0.015
P2 Low Resources	−0.055	0.015
P4 Openness–Imagination	0.295	0.015
P5 Relational Support	0.182	0.014
P6 Comprehensive	0.226	0.014
ESCS	0.238	0.004
Female	0.205	0.009
Immigrant	−0.051	0.011

Note. Reference = P3 (Average-Mixed); *N* = 224,573. Distal means used a BCH classification-error correction (primary), validated against a synthetic benchmark. Uncorrected modal contrasts: 0.233 (P4), 0.146 (P5), 0.206 (P6); population-weighted modal contrasts (0.208, 0.132, 0.174); posterior-probability-weighted means, Openness–Imagination 0.357, Relational Support 0.230, Comprehensive 0.330; unadjusted BCH means, Openness–Imagination 0.434, Relational Support 0.260, Comprehensive 0.330. Standard errors were conditional on the estimated partition. Coefficients are in within-system *SD* units except gender and immigrant background (binary).

Whether the favorable configurations were *equivalent* was tested against the 0.10-*SD* bound after BCH correction (Table 6). The dispositionally rich Openness–Imagination configuration exceeded Relational Support by 0.113 *SD* (90% CI [0.086, 0.140]); this difference was distinguishable from zero, and its interval extended beyond the 0.10-*SD* bound, so equivalence was not established. In contrast, Openness–Imagination and Comprehensive (Δ = 0.069, 90% CI [0.044, 0.094]) and Comprehensive and Relational Support (Δ = 0.044, 90% CI [0.022, 0.066]) were each statistically equivalent within the bound. Thus the dispositionally rich configuration was reliably higher than the relationally supported configuration but not distinguishable from the comprehensively resourced one, and the two contextually distinct configurations that did not differ in dispositions (Comprehensive and Relational Support) performed equivalently.

### 3.5. Which Resources Are Associated with Performance

**Figure 5:**
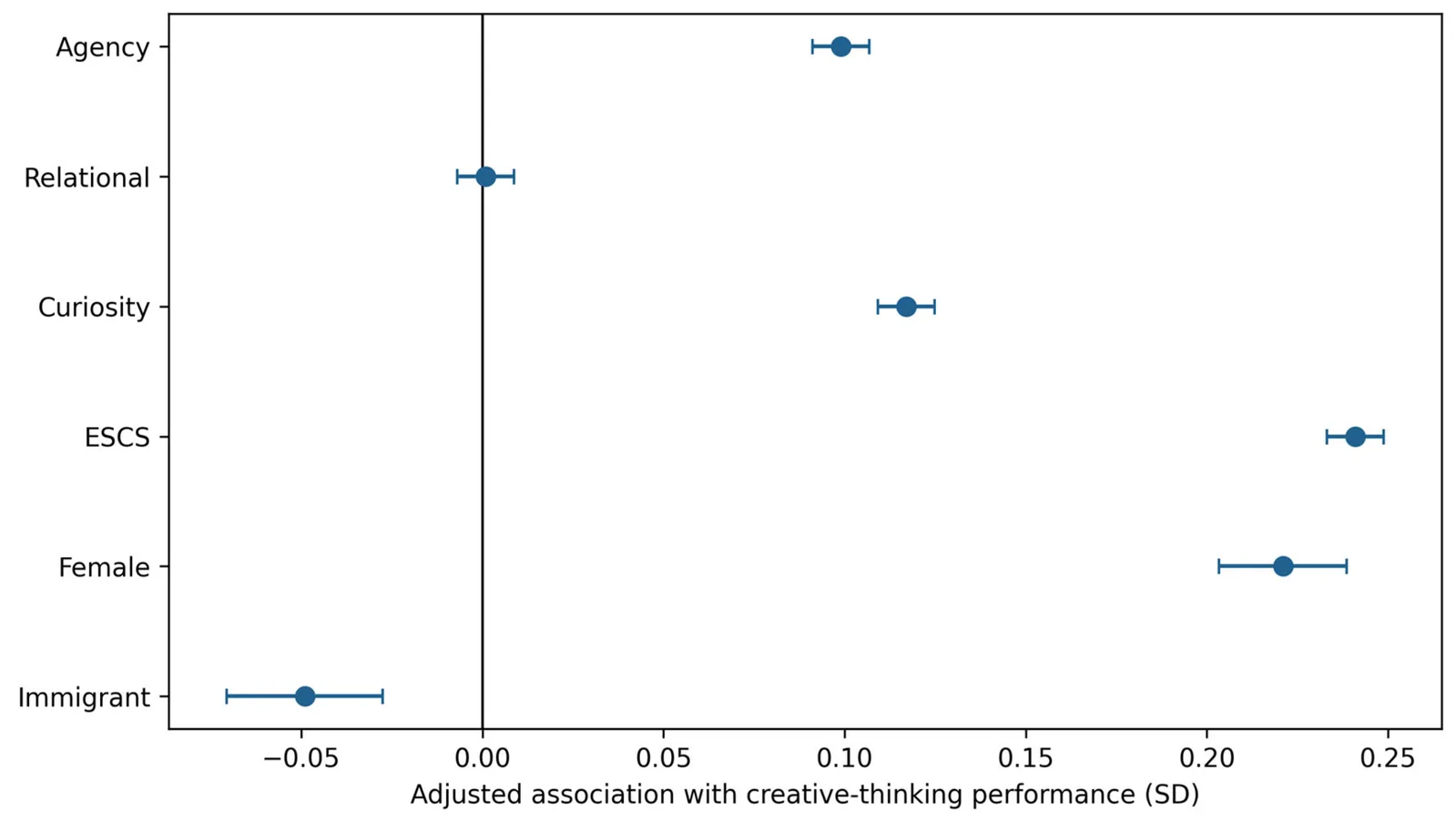
Adjusted associations with creative-thinking performance (restandardized composites and covariates). Points are adjusted coefficients (three resource composites and the ESCS, gender, and immigrant covariates) with 95% conditional Fay BRR confidence intervals (*N* = 224,573). Curiosity and creative agency were positively associated; the relational-support composite was not distinguishable from zero, though it masked opposing indicator-level associations (See Table A3 in Appendix A).

A variable-centered model with restandardized composites related the resources to performance directly (Figure 5; Table 7; *N* = 224,573). Curiosity (0.117) and creative agency (0.099) were positively associated with performance, whereas the relational-support composite was not distinguishable from zero (0.001). Composite *SD*s before restandardization were 0.75, 0.66, and 1.00, and inter-composite correlations ranged from 0.31 to 0.42. A model with the nine individual indicators (See Table A3 in Appendix A) showed that this null composite masked heterogeneous, partly opposing associations: creative-family (0.028) and teacher-support (0.024) were positive, belonging near zero, creative-school negative (−0.051); among dispositions, imagination (0.103) carried most of the agency association.

A within/between-school decomposition (Table 8) indicated that creative agency and curiosity were positively associated with performance at both the student level (within-school 0.104 and 0.096) and, more strongly, the school level (between-school 0.484 and 0.380), whereas relational support was null within schools (−0.001) and slightly negative between schools (−0.061). Because large between-school coefficients can reflect socioeconomic composition, a sensitivity model added within- and between-school ESCS (see Table A4 in Appendix A): school-mean ESCS was itself strongly associated (0.335), and the between-school agency and curiosity coefficients attenuated but remained substantial (0.404 and 0.360), while the between-school relational coefficient attenuated toward zero (−0.030). Because school means based on very few students can be noisy, we re-estimated this model after excluding the 10.7% of schools with fewer than five sampled students (1215 schools): the between-school agency and curiosity coefficients did not attenuate—if anything they were marginally larger (0.432 and 0.404)—so the between-school signal is not an artifact of small-school sampling noise. These are associations in this cross-sectional specification and cannot establish contextual effects; a null estimate for one relational composite does not exclude other contextual mechanisms.

### 3.6. Robustness and Selectivity

Because a single pooled model assigns every student probabilities for the same components, all configurations appeared in every system; per-system recovery was only moderate (congruence 0.83), and several configurations’ prevalence covaried with indicator coverage (Low Resources *r* = −0.77; Openness–Imagination *r* = +0.44). We therefore describe the configurations as estimated in a pooled, within-system standardized sample, not as recurring across systems, pending item-level invariance testing (Cen et al., 2026). Included and excluded (incomplete-indicator) students differed—excluded students scored 0.48 *SD* lower and lower in ESCS—so the complete-case sample is positively selected; nonetheless, the available-data model, which retains these students, reproduced the structure (Tucker φ = 0.998), the outcome ordering, and the adjusted contrasts (above). Raising the coverage threshold to 70%, 80%, and 90% (an eligibility-count exercise) retained 40, 29, and eight systems.

## 4. Discussion

We asked whether creativity-related resources are better represented as discrete profiles or as continua, and, if profiled, whether the profiles are enumerable, reproducible, and useful. The evidence yielded a clear answer with direct implications for how creative ability is measured and modeled.

### 4.1. A Stable but Enumeratively Underdetermined and Predictively Redundant Partition

Given adequate optimization, the six-profile solution was reproducible—recovered in most restarts and highly stable across subsamples (ARI = 0.95). Reproducibility, however, did not establish sufficiency. No enumerative optimum emerged within the tested range, and, notably, the profiles carried negligible out-of-sample predictive value under leakage-safe cross-validation: at no resolution from *K* = 2 to *K* = 12, and whether entered as modal classes or posterior probabilities, did profile prediction approach a flexible continuous model, to which profiles added a mean incremental *R*^2^ of only 0.004 (below the 0.005 negligibility threshold). The profiles’ modest increment was consistent with discretized nonlinearities that a continuous specification captures directly. This finding carries a specific methodological implication: a latent-profile solution can be stable and interpretable yet remain an enumeratively underdetermined, predictively redundant re-description of continuous variation (Bauer & Curran, 2003). Person-centered “types” should be reported only alongside a selected class count, reproducibility evidence, and incremental value benchmarked against a flexible continuous model. We intend a precise reading of this result. It shows that, for this outcome, profile representations were predictively redundant and that the data did not select a unique class count within *K* = 2–12; it does not show that discrete latent subpopulations are absent in any ontological sense. Lack of incremental predictive utility and enumerative underdetermination are claims about the usefulness and identifiability of a discrete representation, not about the metaphysical existence of latent classes, which a purely predictive and information-criterion analysis cannot adjudicate.

### 4.2. Outcome Associations: A Partial Dispositional Ordering

Read as descriptive strata, the three resource-favorable configurations showed above-average performance, and their ordering—Openness–Imagination highest—recurred in every subsample. After classification-error correction, the dispositionally rich configuration was reliably higher than the relationally supported configuration (by about 0.11 *SD*, beyond the 0.10-*SD* bound) but was not distinguishable from the comprehensively resourced configuration, and the comprehensively resourced and relationally supported configurations performed equivalently. That a configuration with no greater average resource level than Relational Support (Openness–Imagination) nonetheless performed higher, whereas the higher-resource Comprehensive configuration did not exceed it, points to the differential relevance of specific resources rather than a cumulative count, and is inconsistent with a strong, equal-outcome reading of equifinality applied to all favorable configurations. Because the design is cross-sectional, none of these findings addresses developmental routes.

### 4.3. A Proximal Dispositional Signal

The variable-centered, individual-indicator, and within/between-school analyses converged on creative dispositions and curiosity rather than perceived relational context, and localized the signal to imagination and curiosity. The between-school decomposition showed that agency and curiosity were associated with performance at the school level even after adjusting for school-mean ESCS, whereas relational support was not; this speaks against the possibility that a student-level model had obscured a positive relational contextual association, although the cross-sectional decomposition cannot establish contextual effects and a null for one relational composite does not exclude other mechanisms. This interpretation should remain cautious: creative self-efficacy, openness, imagination, and curiosity are conceptually and measurement-proximal to a creative-thinking test, and PISA’s scores partly index general cognitive engagement (Kraus et al., 2026), so larger contemporaneous coefficients are partly expected on those grounds. The negative coefficient for perceived creative school environment deserves comment, because at face value one would expect a more creativity-encouraging school to foster creative thinking. Several non-substantive mechanisms can produce this sign, and they are not mutually exclusive. First, the indicator is a *perception* rather than an objective feature of instruction, and perceptions are calibrated against a local standard: in schools where creative activity is more salient, students may judge any given level of encouragement more critically, a reference-group (big-fish–little-pond) effect (Marsh & Parker, 1984) that can invert the individual-level sign, consistent with the negative *between-school* coefficient we observed. Second, the item may capture reporting style and acquiescence as much as classroom reality, and shared-method variance with the other self-reports can distort partial coefficients once the correlated agency indicators are held constant. Third, schools that emphasize creative activities may do so partly in response to lower baseline performance, producing a negative cross-sectional association that reflects selection rather than a causal effect of climate. Because the relational composite has modest internal coherence (*α* = 0.59) and pools four heterogeneous sources, its near-zero aggregate coefficient masks precisely these offsetting indicator-level signals; we therefore interpret the relational results at the indicator level and treat the negative creative-school coefficient as a hypothesis about perception and selection rather than evidence that creativity-supportive schooling depresses performance. A defensible statement is narrow: performance-proximal creative dispositions and curiosity were more strongly associated with assessed creative thinking, at both the student and the school level, than the measured relational perceptions.

### 4.4. Implications for the Measurement and Modeling of Creative Ability

Beyond the lesson about latent-profile analysis, the results bear on how creative ability and its correlates should be measured and modeled. Conventional cognitive abilities are represented dimensionally—as continuous latent factors—precisely because graded individual differences carry the information that predicts criteria and supports validity work (Carroll, 1993). Our findings extend the same logic to the resources associated with creative-thinking performance. A flexible continuous model predicted performance markedly better than any discrete partition, and converting the indicators into profiles discarded predictive information at every resolution tested. Discrete “types” collapse the graded standing that dimensional models retain, impose a class count the data did not select, and are not obviously reproducible across systems without invariance testing. Continuous representations, by contrast, preserve rank-order information, integrate naturally with latent-variable and multilevel measurement models, and permit measurement-invariance testing before any cross-national comparison. For the science of creative ability, then, modeling creativity resources as continuous latent dimensions—ideally within invariance-tested, multilevel measurement models—is more scientifically informative, and more reproducible, than sorting students into resource “types.” Substantively, assessed creative thinking behaved as a cognitive individual-difference variable: it was predicted most by performance-proximal dispositions such as imagination and curiosity, consistent with its partial overlap with general cognitive engagement (Kraus et al., 2026) even as it remains conceptually distinct from conventional intelligence (Nusbaum & Silvia, 2011; Silvia, 2015).

### 4.5. Implications for Educational Practice and Assessment

Two applied recommendations follow, offered with the caution that the design is cross-sectional. First, for screening, progress monitoring, and research use, creativity-related resources are better recorded and modeled as continuous indicators than as student “types.” Sorting students into a small number of resource profiles discarded predictive information at every resolution we tested and imposed a class count the data did not support. Retaining the graded indicators preserves the information that predicts performance and avoids the risk that a convenient label—“the low-resource student”—is reified as a stable kind and used to route instruction. Where a compact summary is needed for communication, a profile may still be reported descriptively, but it should not be the unit on which decisions about students are based.

Second, the associations—though modest and non-causal—point interventions toward the most performance-proximal and plausibly malleable dispositions, curiosity and imagination, rather than toward broad relational-climate perceptions, whose aggregate had no detectable association with assessed performance and whose components pulled in opposing directions. Programs that cultivate curiosity and imaginative exploration therefore have a clearer empirical rationale here than efforts targeting generalized perceptions of a “creative school.” The counterintuitive negative coefficient for perceived creative school environment reinforces a practical caution: self-reported climate indicators are calibrated against local expectations and are easily misread, so they are better treated as descriptive context than as targets or as indicators of program success. None of these points should be read causally; they indicate only where continuous measurement and proximal, malleable targets are most defensible on present evidence, and they motivate the longitudinal and experimental work outlined next.

### 4.6. Limitations and Future Directions

The study is cross-sectional; configurations are patterns of co-occurring self-reports, not developmental routes. Outcome standard errors are conditional on the estimated partition. The complete-case sample is positively selected, though the available-data model reproduced the conclusions. Student-level item measurement invariance was not tested, and prevalence partly covaried with coverage (Cen et al., 2026). The indicators are self-reports, and the assessment mixes creativity with general skill and effort (Kraus et al., 2026). A diagonal covariance structure was assumed; a fuller covariance structure might alter the class solution. The within/between-school analysis is a fixed decomposition, not a random-effects model; a full multilevel latent-variable model with richer composition controls would sharpen it. Future work should test configural and metric invariance before cross-system profile comparison, model creativity resources as continuous latent variables in a multilevel framework, and use longitudinal and experimental designs to test developmental or causal claims.

## 5. Conclusions

Given adequate optimization, creativity-related resource profiles in PISA 2022 were reproducible, but no class count was selected by the data and the profiles added negligible out-of-sample predictive value—at any resolution and under soft or hard classification—beyond the continuous dispositions and contexts from which they were built; a flexible continuous model predicted creative-thinking performance better. Read as descriptive strata, and after a validated classification-error correction, a dispositionally rich configuration was reliably higher than a relationally supported configuration but not distinguishable from a comprehensively resourced one, and performance-proximal dispositions and curiosity were the resources most associated with performance. For the measurement and modeling of creative ability, these results argue for continuous, dimensional representations over discrete student “types,” and for reporting class enumeration, reproducibility, and incremental value—benchmarked against a flexible continuous model—before any latent-profile solution is interpreted substantively.

## Figures and Tables

**Table 1:** Analytic sample and design characteristics.

Characteristic	Value
Systems in questionnaire file	80
Systems administering creative-thinking assessment (in file)	63
Eligible systems (analysis)	44
Students (available data)	306,704
Complete-indicator students (primary)	235,240
Schools	11,620
Female students	50.4%
Immigrant-background students	12.5%
ESCS, *M* (*SD*)	−0.24 (1.07)
Complete cases (all 9 indicators)	76.7%
Minimum pairwise joint coverage	80.8%

Note. Eligibility required ≥ 500 students with a creative-thinking plausible value and ≥50% coverage on every indicator. Ukraine (released separately) is absent from this file. The 44 systems and full eligibility matrix are in the Appendix A.

**Table 2:** Class enumeration, *K* = 2–12 (senate-weighted, unit-mean scale).

K	Starts	AIC	BIC	Entropy	Smallest Class	Best-LL Recovery (±5)
2	10	1,826,466	1,826,813	0.717	42.5%	100%
3	10	1,772,565	1,773,091	0.751	15.3%	100%
4	25	1,751,366	1,752,070	0.696	13.4%	84%
5	25	1,734,588	1,735,470	0.685	12.0%	28%
6	50	1,723,572	1,724,633	0.674	11.2%	60%
7	25	1,714,712	1,715,951	0.676	4.7%	28%
8	25	1,709,100	1,710,517	0.656	4.3%	4%
9	15	1,704,173	1,705,769	0.656	4.1%	13%
10	8	1,700,722	1,702,496	0.673	1.3%	12%
11	8	1,695,649	1,697,601	0.660	1.2%	38%
12	7	1,692,555	1,694,686	0.651	1.2%	14%

Note. System-balanced enumeration sample (≤2000 students/system; pseudo-likelihood *N* = 88,000), senate weights normalized so each system contributes *N*/*C*. AIC and BIC declined monotonically, so no class count was selected within the tested range. Entropy and “best-LL recovery” (percentage of starts within 5 log-likelihood units of the best solution) pertain to this balanced enumeration sample; the working six-profile solution (complete-case sample) had entropy of 0.755.

**Table 3:** Reproducibility and robustness summary.

Diagnostic	Result
Best six-profile log-likelihood recovery (50 starts, ±5; enumeration sample)	60%
ARI to pooled solution (12 repeated subsamples)	*M* = 0.95, range 0.88–0.97
Outcome ordering (Openness–Imagination highest) recurrence	12 of 12 subsamples
Complete-case vs. available-data classification (ARI)	0.92
Complete-case vs. available-data profile shape (Tucker φ)	0.998
Independent per-system congruence to pooled (Tucker φ)	*M* = 0.83, range 0.63–0.97 (*r* with *n* = 0.26)
Excluding Albania: congruence to full	1.000 (ordering unchanged)
Excluding 7 sampling-caution systems: congruence to full	0.999 (ordering unchanged)
Class-varying vs. equal variances (BIC, full sample)	5.20 M vs. 5.47 M (class-varying preferred)
Within-class residual correlations	max |*r*| = 0.22, *M* = 0.06

Note. ARI = adjusted Rand index; φ = Tucker’s congruence coefficient. Repeated subsamples were drawn independently and could overlap; they were not independent replications.

**Table 4:** Leakage-safe cross-validated variance explained (school-grouped, repeated 3 × 5-fold, best of four starts per fold).

Model	CV *R*^2^ (*M*)	Range Across Folds
Nine continuous indicators (linear)	0.044	0.027–0.061
Flexible continuous (indicators + quadratic + interactions)	0.068	0.051–0.083
Six profile dummies (modal)	0.028	0.017–0.038
Six profiles (posterior probabilities)	0.034	0.022–0.046
Continuous (9 linear) + profiles	0.053	0.036–0.069
Flexible continuous + profiles	0.072	0.057–0.087

Note. Fifteen folds (3 repetitions × 5 folds), folds assigned by school within system; the mixture (best of four random starts; mean recovery 2.9 of 4) and standardization were refitted within each training fold. Prediction models used the resource representation only (no covariates). The mean incremental Δ*R*^2^ of profiles over the flexible model was 0.004 (repetition-level means 0.004, 0.004, 0.004), below the 0.005 negligibility threshold; ranges are descriptive because folds sharing training data are dependent. Prediction across *K* = 2–12 appears in Figure 2.

**Table 6:** Pairwise differences and equivalence tests among the favorable configurations (BCH-Corrected).

Contrast	Δ	*SE*	90% CI	TOST (Lower/Upper)	Conclusion
Openness–Imagination vs. Relational Support	0.113	0.016	[0.086, 0.140]	<0.001/0.78	Different from 0; CI exceeds 0.10 bound
Openness–Imagination vs. Comprehensive	0.069	0.015	[0.044, 0.094]	<0.001/0.02	Equivalent within 0.10
Comprehensive vs. Relational Support	0.044	0.013	[0.022, 0.066]	<0.001/<0.001	Equivalent within 0.10

Note. SESOI = 0.10 *SD* (chosen a priori; study not preregistered). TOST = two one-sided tests; “lower” tests Δ ≤ −0.10, “upper” tests Δ ≥ +0.10. Openness–Imagination exceeded Relational Support by a margin whose interval extended beyond the 0.10-*SD* bound (equivalence not established); the other two contrasts were equivalent within the bound.

**Table 7:** Variable-centered model, restandardized composites (conditional Fay BRR).

Predictor	*b*	*SE*
Creative agency (restandardized)	0.099	0.004
Relational support (restandardized)	0.001	0.004
Curiosity	0.117	0.004
ESCS	0.241	0.004
Female	0.221	0.009
Immigrant	−0.049	0.011

Note. *N* = 224,573. Composites restandardized within system so coefficients are per-*SD* comparable (pre-restandardization *SD*s: agency 0.75, relational 0.66, curiosity 1.00; inter-composite *r* = 0.31–0.42).

**Table 8:** Within- and between-school decomposition of the composites (conditional Fay BRR).

Predictor	*b*	*SE*
Creative agency, within-school	0.104	0.005
Creative agency, between-school	0.484	0.026
Relational support, within-school	−0.001	0.006
Relational support, between-school	−0.061	0.029
Curiosity, within-school	0.096	0.004
Curiosity, between-school	0.380	0.020
ESCS	0.226	0.004
Female	0.197	0.009
Immigrant	−0.059	0.011

Note. *N* = 224,573. Each composite was decomposed into its student-final-weight-weighted school mean (between-school) and the within-school deviation. Coefficients are associations in this fixed decomposition, not multilevel-model effects. A sensitivity model adding within/between-school ESCS is in Table A4 in the Appendix A.

## Data Availability

The data analyzed in this study are publicly available from the OECD PISA 2022 database (https://www.oecd.org/pisa/data/ (accessed on 16 August 2026); file CY08MSP_STU_QQQ.SAV).

## Supplementary Materials

The following supporting information can be downloaded at: https://www.mdpi.com/article/10.3390/jintelligence14090207/s1, an anonymized analytic-code archive with a configurable data path, an environment specification (requirements.txt), an ordered runner (RUN_ORDER.md), and the derived result files that reproduce every reported table and figure. The additional sensitivity analyses reported in the text—senate-weighted standardization congruence, exclusion of schools with fewer than five sampled students, composite inter-item correlations, per-fit EM iteration counts, and BCH classification-matrix conditioning—are reproduced by the same code. Additional Table A1, Table A2, Table A3 and Table A4 are provided in Appendix A.

## Appendix A. Tables

**Table A1:** Pathway indicators (nine OECD WLE indices).

Index	Construct	Composite	Direction	Coverage Range
CREATEFF	Creative self-efficacy	Agency	Higher = more	0.65–0.99
CREATOP	Creativity/openness to intellect	Agency	Higher = more	0.62–0.99
IMAGINE	Imagination and adventurousness	Agency	Higher = more	0.59–0.99
OPENART	Openness to art and reflection	Agency	Higher = more	0.60–0.99
CREATFAM	Creative family environment	Relational	Higher = more	0.62–0.99
CREATSCH	Creative school environment	Relational	Higher = more	0.62–0.99
BELONG	Sense of belonging	Relational	Higher = more	0.88–10.00
TEACHSUP	Teacher support	Relational	Higher = more	0.78–0.99
CURIOAGR	Curiosity	(standalone)	Higher = more	0.78–0.99

Note. WLE = weighted likelihood estimate. Composites are unit-weighted means of within-system standardized indicators, restandardized within system. Item counts and per-system reliabilities are tabulated in the PISA 2022 technical report (OECD, 2024c); the underlying item responses, available in the public database, were not loaded for this analysis. Coverage range is the min–max proportion with a valid value across systems.

**Table A2:** Configuration characteristics and outcomes (complete-case sample).

Profile	Label	%	Mean Resource *z*	Creative *z* (SE)
P1	Constrained	10.9	−0.69	−0.30 (0.009)
P2	Low Resources	27.4	−0.39	−0.01 (0.008)
P3	Average-Mixed	14.1	0.02	0.06 (0.009)
P4	Openness–Imagination	15.7	0.19	0.38 (0.008)
P5	Relational Support	18.6	0.19	0.24 (0.008)
P6	Comprehensive	13.3	0.96	0.33 (0.009)

Note. Percentages are senate-weighted posterior prevalence. Creative *z* is mean modal-class performance standardized within system, pooled across 10 plausible values. P4 and P5 had a similar average indicator level yet differed in performance.

**Table A3:** Variable-centered model with the nine individual indicators (conditional Fay BRR).

Indicator	*b*	*SE*
Creative self-efficacy	0.011	0.004
Openness to intellect	0.038	0.004
Imagination	0.103	0.004
Openness to art	−0.009	0.003
Creative family environment	0.028	0.003
Creative school environment	−0.051	0.004
Belonging	0.011	0.003
Teacher support	0.024	0.004
Curiosity	0.112	0.004

Note. *N* = 224,573; adjusted for ESCS, gender, and immigrant background (omitted). The null relational composite (Table 7) masked opposing indicator-level associations: creative-family and teacher-support positive, creative-school negative. Imagination and curiosity were the strongest indicator-level correlates.

**Table A4:** Within/between-school decomposition adding school-mean ESCS (composition sensitivity; conditional Fay BRR).

Predictor	*b*	*SE*
Creative agency, within-school	0.113	0.005
Creative agency, between-school	0.404	0.025
Relational support, within-school	0.001	0.006
Relational support, between-school	−0.030	0.029
Curiosity, within-school	0.100	0.004
Curiosity, between-school	0.360	0.020
ESCS, within-school	0.143	0.004
ESCS, between-school	0.335	0.006
Female	0.196	0.009
Immigrant	−0.095	0.011

Note. *N* = 224,573; 11,369 schools (median 20 students/school; 10.7% with <5 students). Adding within/between-school ESCS, the between-school agency and curiosity coefficients attenuated (from 0.484 and 0.380 in Table 8 to 0.404 and 0.360) but remained substantial, whereas the between-school relational coefficient attenuated toward zero. Coefficients are associations in this fixed decomposition.

Included systems (44 PISA codes): ALB, ARE, AUS, BGR, BRA, CHL, COL, CRI, CZE, DNK, EST, FIN, FRA, GRC, HKG, HRV, HUN, IDN, ISL, ITA, JAM, JOR, KOR, LTU, LVA, MKD, MLT, MNG, NLD, NZL, POL, PRT, PSE, QAT, QUR, ROU, SAU, SLV, SRB, SVK, SVN, TAP, URY, UZB.

Sample flow: The file (CY08MSP_STU_QQQ.SAV) contained 80 systems and 613,744 students; Ukraine, released separately, is the system absent relative to the 81 commonly reported participants. Sixty-three systems in the file had creative-thinking plausible values. Nineteen were excluded for coverage below 50%—all because they did not field the creativity-questionnaire scales (coverage ≈ 0): BEL, BRN, CAN, DEU, DOM, ESP, ISR, KAZ, MAC, MAR, MDA, MEX, MYS, PAN, PER, PHL, QAZ, SGP, THA. A machine-readable eligibility matrix accompanies the code. All mixture estimation and cross-validation used the senate-scaled final student weight; the 80 Fay replicate weights were used only for conditional outcome inference. Anonymized code with fixed seeds and an environment specification reproduces every table and figure and contains no usernames, local paths, or author metadata.
